# Implementation methods of infection prevention measures in orthopedics and traumatology – a systematic review

**DOI:** 10.1007/s00068-020-01477-z

**Published:** 2020-09-10

**Authors:** Benedikt Marche, Meike Neuwirth, Christiane Kugler, Bertil Bouillon, Frauke Mattner, Robin Otchwemah

**Affiliations:** 1 Dept. of Orthopedics, Trauma Surgery and Sports Medicine, University Witten/Herdecke, Cologne Merheim Medical Center, Ostmerheimer Str. 200, 51109 Cologne, Germany; 2 Institute for Hygiene, Cologne Merheim Medical Center, University Hospital University Witten/Herdecke, Ostmerheimer Str. 200, 51109 Cologne, Germany; 3 Bereich Pflegewissenschaft, Albrecht-Ludwigs-Universität Freiburg, Freiburg, Germany

**Keywords:** Infection prevention, Implementation, Surgical site infection, Perioperative management

## Abstract

**Background:**

Prevention of hospital-acquired infections, in the clinical field of orthopedics and traumatology especially surgical site infections, is one of the major concerns of patients and physicians alike. Many studies have been conducted proving effective infection prevention measures. The clinical setting, however, requires strategies to transform this knowledge into practice.

**Question/purpose:**

As part of the HYGArzt-Project (“Proof Of Effectivity And Efficiency Of Implementation Of Infection Prevention (IP) Measures By The Physician Responsible For Infection Prevention Matters In Traumatology/Orthopedics”), the objective of this study was to identify effective implementation strategies for IP (infection prevention) measures in orthopedics and trauma surgery.

**Methods:**

The systematic review was conducted following PRISMA guidelines. A review protocol was drafted prior to the literature search (not registered). Literature search was performed in MEDLINE*,* SCOPUS and COCHRANE between January 01, 1950 and June 01, 2019. We searched for all papers dealing with infection and infection control measures in orthopedics and traumatology, which were then scanned for implementation contents. All study designs were considered eligible. Exclusion criteria were language other than English or German and insufficient reporting of implementation methods. Analyzed outcome parameters were study design, patient cohort, infection prevention measure, implementation methods, involved personnel, reported outcome of the studies and study period.

**Results:**

The literature search resulted in 8414 citations. 13 records were eligible for analysis (all published between 2001 and 2019). Studies were primarily prospective cohort studies featuring various designs and including single IP measures to multi-measure IP bundles. Described methods of implementation were heterogeneous. Main outcome parameters were increase of adherence (iA) to infection prevention (IP) measures or decrease in surgical site infection rate (dSSI%). Positive results were reported in 11 out of 13 studies. Successful implementation methods were building of a multidisciplinary team (considered in 8 out of 11 successful studies [concerning dSSI% in 5 studies, concerning iA in five studies]), standardization of guidelines (considered in 10/11 successful studies [concerning dSSI% in 5 studies, concerning iA in seven studies]), printed or electronic information material (for patient and/or staff; considered in 9/11 successful studies [concerning dSSI% 4/4, concerning iA 5/5]), audits and regular meetings, personal training and other interactive measures as well as regular feedback (considered in 7/11 successful studies each). Personnel most frequently involved were physicians (of those, most frequently surgeons) and nursing professions.

**Conclusion:**

Although evidence was scarce and quality-inconsistent, we found that adhering to a set of implementation methods focusing on interdisciplinary and interactive /interpersonal work might be an advisable strategy when planning IP improvement interventions in orthopedics and traumatology.

**Electronic supplementary material:**

The online version of this article (10.1007/s00068-020-01477-z) contains supplementary material, which is available to authorized users.

## Background/introduction

Prevention of hospital-acquired infections is one of the major concerns of patients and physicians alike. Perioperative infections in the clinical field of orthopedics and traumatology pose a huge obstacle as the consequences in case of osteitis and implant failure can be devastating [1–3]. Implant-related infections usually require long-term antibiotic therapy with possible development of resistances and possibly multiple surgical revisions [4, 5]. In addition to clinical outcomes, the negative socioeconomic implications are immense [5–7]. There has been an increasing number of publications concerning successful single and bundled infection prevention measures in recent years. Those summarizing, the *World Health Organization* published a guideline on preventive measures of surgical site infections in 2016 [8]. In addition, reviews on specific IP measures in the clinical field of orthopedics and traumatology were published which surpassed the WHO recommendations [9, 10]. Although in many cases, we do theoretically know which IP measures are associated with the best outcome, the measures themselves are often carried out insufficiently in practice [11, 12]. The question remains: Which methods/strategies to implement those proven IP measures into our clinical practice work best? The relatively young field of implementation science aims to find scientifically proven ways to transmit desired operations into practice [13].

## Purpose

As part of the *HYGArzt-*Project (“*Proof Of Effectivity And Efficiency Of Implementation Of Infection Prevention (IP) Measures By The Physician Responsible For Infection Prevention Matters In Traumatology/Orthopedics” *Grant Number ZMVI1‐2516FSB111), the objective of this study was to systematically review and identify effective implementation methods of IP measures in orthopedics and traumatology.

## Materials and methods

### Search strategy

**Fig. 1:**
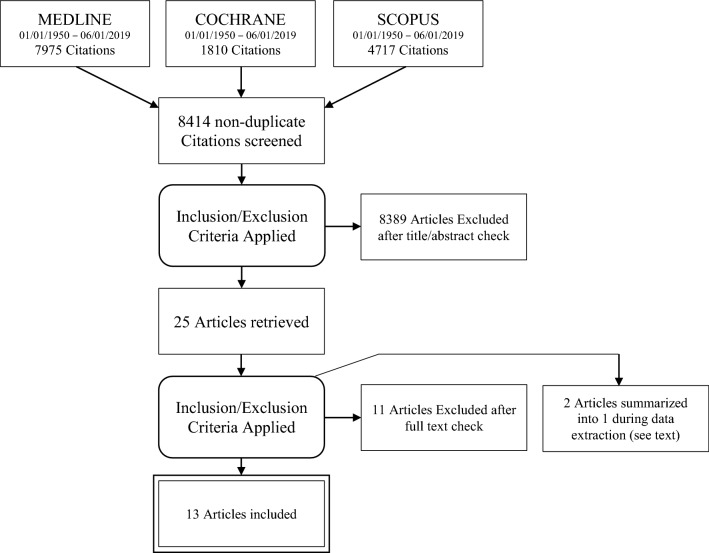
PRISMA flow diagram of screening process for the systematic review of the literature for implementation methods of infection prevention measures in orthopedics and traumatology

A systematic literature review was conducted following PRISMA (Preferred Reporting Items for Systematic Reviews and Meta-analyses) statement guidelines [14]. The literature search was performed in the databases *MEDLINE, SCOPUS* and *COCHRANE LIBRARY* including articles published between 01/01/1950 and 06/01/2019, using the terms “[(infection AND prevention) OR implementation OR hygiene OR (quality AND improvement)] AND (orthopedics OR traumatology OR (sports AND medicine) OR arthroplasty)”. Cluster searches were performed when relevant literature was encountered. For the flow chart, see Fig. 1.

### Study screening

Two reviewers independently screened the titles, abstracts and full-text articles in duplicate. The reviewers discussed all discrepancies to reach a consensus. The references of the included studies were subsequently searched by the reviewers to manually identify any articles that may have eluded the initial search.

### Assessment of study eligibility

The inclusion criteria were as follows: Studies which reported the implementation methods of IP measures in the clinical field of orthopedics and traumatology; language German or English; all human study populations; all publication dates; all types of outcome parameters; studies of all levels of evidence. Any types of hospital-acquired infection in the field of orthopedics and traumatology were included. Primarily, all types of research or studies were considered eligible. We agreed on including NRSI (non-randomized studies on intervention effects), as the answer to the review question does not necessarily rely on RCT-study designs. Exclusion criteria were: articles of editorial type, book chapters; language other than English or German; patient collective other than orthopedic and/or traumatological; insufficient reporting of implementation methods. At least one procedural step undertaken to implement the desired IP measure had to be described.

### Assessment of study quality

The MINORS (Methodological Index for Non-randomized Studies) checklist was used to assess the methodological quality of the included prospective interventional cohort studies [15]. CONSORT statement for the reporting of cluster-randomized trials (2010) was consulted for assessment of the included cluster RCT [16]. Both reviewers independently scored the included studies and discussed differences in scoring until consensus was achieved. Risk of bias assessment for the non-randomized studies was performed using seven domains from a modified ROBINS-I-tool.

### Data extraction and analysis

The resulting studies were searched for study design, patient cohort, types of IP measures implemented, outcome parameters, outcome(s), implementation method(s), personnel involved and study periods. Microsoft Excel^®^ Version 16 was used for the calculations and graphics. Meta-analysis was not planned and not performed. Data extraction was performed by the reviewers independently, especially in regard to the summarisation of implementation methods.

## Results

### Literature search, study screening

The literature search resulted in 8414 articles. After applying inclusion and exclusion criteria in title and abstract check as stated above, 26 records were eligible for full-text analysis. After the full-text analysis with exclusions as listed in Supplement 1, *n* = 14 studies were included in the Review of implementation methods for IP measures in orthopedics and traumatology. Two included studies dealt with the same QI (quality improvement) project, and after contacting the authors and confirmation of 100% overlapping of study population, the two reviewers agreed on combining the study data and regarding the two studies as one [17, 18]. Thus, the resulting number of included studies was 13 (Table 1; detailed view Supplement 2). No relevant inter-observer difference on study inclusion was present. No published (systematic) review on the topic could be identified so far.

**Table 1:** Implementation methods

Study/year	IP MEASURE (Single measure or bundle strategy)	Implementation methods								
		Development of standardized	Printed materials	Multidisciplinary team	Audits/regular meetings	Personal training/lectures	Feedback	Online/electronic materials	Other	Outcome
Douglas 2001	Bundle	■	■	■	■	■				Successful (reduced SSI%, reduced LOS)
Queiroz 2005	Single	■		■		■		■	■	Successful (reduced SSI%, increased A/C, reduced COA and DDD)
Macdonald 2006	Single		■			■	■			Successful (increased surface area of hand disinfection)
McCahill 2007	Single	■	■	■	■		■		■	Successful (increased A/C)
Rosenberg 2008	Single	■	■						■	Successful (increased A/C)
Nobile 2014	Single	■	■	■	■	■	■	■	■	Successful (increased A/C)
Yang 2014	Single	■						■		Successful (increased A/C, reduced LOS and COS, no change in SSI%)
Khodyakov 2015	Bundle	■	■	■	■	■	■	■	■	Successful (increased A/C)
Mori 2015	Bundle	■	■	■	■	■	■		■	Successful (reduced SSI%)
Shea 2015	Bundle	■	■	■	■	■	■	■	■	Successful (reduced SSI%)
Schriefer 2017	Bundle	■	■	■	■	■	■	■	■	Successful (reduced SSI%, increased A/C)
Mackain-Bremner 2008	Bundle		■							Not successful (no increase in adherence)
Kapadia 2015	Single		■			■			■	Not successful (low adherence)
TOTAL implementation method included in		10	8	7	7	8	7	6	8	out of 10 studies reporting success

*IP* infection prevention; *SSI%* surgical site infection rate; *LOS* length of stay; *A/C* adherence/compliance; *COA* cost of antibiotics; *DDD* defined daily doses of antibiotics)

### Quality of studies

The MINORS scores for the 10 prospective cohort studies ranged from 4/16 points to 13/16 points. On average, the MINORS score was 9.3/16. The included non-randomized comparative prospective cohort study was scored 18/24 in the MINORS score [19]. There were no relevant inter-observer differences on MINORS scores (Kappa value not calculated). Individual MINORS scores can be seen in Supplement 3. Risk of Bias assessment of the included non-randomized studies, as summarized in Supplement 4, showed varying results, overall Risk of bias has to be valued as moderate to high. Deficits were mostly found across the domains: inconsistent reporting of study population and inclusion criteria, and outcome parameters, inconsistent (a-priori-calculation of) sample sizes/power calculations, inconsistent reporting of statistical analysis. Follow-up data to determine the sustainability of the intervention were also not consistently reported. Descriptions of hospital data and staff responsibilities were not consistently reported. The sets of baseline parameters showed high variance (i.e., SSI rates ranging from 30 to 4%) throughout the studies. Outcome measurements and reporting were found to be at risk of a bias. The included cluster-randomized trial was of excellent quality, completely satisfying the CONSORT statement criteria for cluster-randomized trials of 100% with a low risk of bias [17, 18].

Information on funding of the included studies was not provided in 5 out of 13 studies; public funding from Health Authority was stated in 3 out of 13 studies. 3 out of 13 studies stated absence of external funding. Two studies stated author affiliation to health industry including external funding.

### Data extraction and analysis

#### Study designs, patient cohort

Date of publication of the included studies ranged from 2001 to 2017 [20, 21].

Studies were primarily prospective cohort studies (“pre–post studies”, 10 out of 13 studies) featuring various designs from single interventions to multi-measure bundled interventions. One cluster-randomized trial was included [17, 18]. One study directly compared two different methods of implementation (no information provided if randomization was performed) [19]. Most studies (11 out of 13 studies) sampled a pre-interventional baseline set of parameters (e.g., SSI rate or adherence to certain IP measures) and reported the postinterventional re-measurements. One study delivered no pre-intervention data and was of retrospective observational type [22]. The patient cohort was exclusively “elective orthopedics” in 10 out of 13 studies. One study also included traumatologic patients/emergent surgery [23]; two studies dealt with pediatric orthopedic patients [21, 24]. Two studies did not further specify their observed cohort and stated “orthopedic” [19, 25]. Reporting of sample size was inconsistent. In 7 out of 13 studies, the sample size was stated as a number of patients or surgeries. Mean sample size of the prospective studies was 331 patients or surgeries (range 124–717), the retrospective study included *n* = 4751 surgeries. 2 out of 13 studies reported a sample size as a number of involved staff members (*n* = 55 and *n* = 82) [25, 26]. One study stated a sample size as a number of hospitals (*n* = 188 hospitals) [17, 18]. It has to be noted that this last-mentioned study was a report on a multi-hospital Quality Improvement campaign in contrast to all other 12 out of 13 studies, which were single-centered. 3 out of 13 studies did not report any sample size [20, 24, 27]. Contacting the authors provided no further information. Patient demographics and characteristics, such as preconditions, were reported in 1 out of 13 studies [19].

#### Types of IP measures implemented

More than half of the included studies (7 out of 13 studies) included a single IP measure. The other 6 out of 13 studies used a bundled approach of more than one IP measure.

Single measures included: Correct delivery of PAP (perioperative antibiotic prophylaxis) in 5 out of 7 studies. One study investigated hand washing performance of the orthopedic staff; one study investigated preoperative antiseptic washing by the patient [22, 26].

**Fig. 2:**
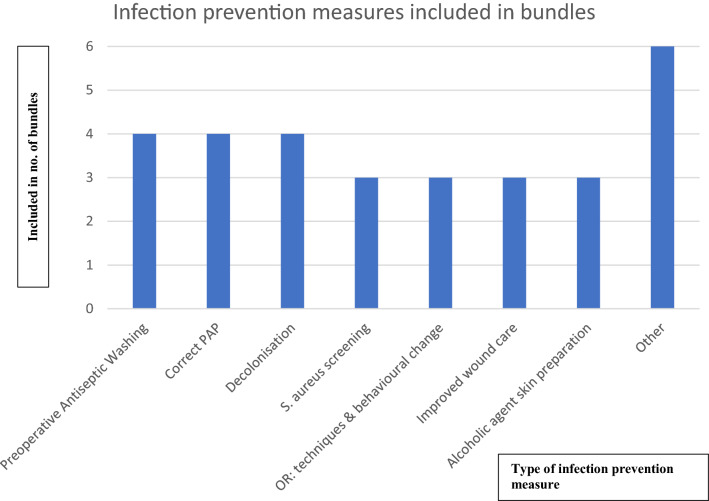
Infection prevention measures included in bundles. *OR* operation room; other: Risk stratification of Patients, Urinary catheter discontinuation, Preoperative nutrition management, perioperative normothermia, hair removal technique

Bundled infection prevention measures approaches (6 out of 13 studies) included on average 4.7 different IP measures (range 3–9) (Fig. 2). Interventions to change different aspects of behavior in the OR (operating room) were rated as one IP measure (e.g., covering of hair, covering of jewelry, minimizing traffic, tucking in of shirts, minimizing noise, etc.).

#### Outcome parameters

The main outcome parameters in the studies were measurement of change in adherence to the desired IP measure (iA; reported in 9 out of 13 studies) and change in SSI rate (SSI%; reported in 6 out of 13 studies); others included Length Of Stay (LOS; reported in 2 out of 13 studies), Cost Of Antibiotics Per Case (reported in 2 out of 13 studies) and defined daily doses of antibiotics per 100 bed-days (DDD; reported in 1 out of 13 studies). One study measured missed moistened surface area of hands (%) in a hand hygiene study [25]. One study reported use rate of provided implementation resources [17, 18].

Adherence was consistently measured as the number of times the desired IP measure was carried out (or rather carried out correctly and in the indicated situations) by the target population.

There were no outcome parameters concerning infection rates other than SSI (i.e., pneumonia or uniary tract infections).

#### Outcomes

Two out of the 13 included studies reported negative results: Mackain-Bremner et al. reported no significant change in adherence after the intervention (bundled behavioral IP measures), valuing their intervention as unsuccessful [26]. Kapadia et al., in a retrospective study of a single IP measure (preoperative antiseptic washings by patients), did not report a change in compliance due to a lack of baseline parameters/control group but valued the overall compliance of 22% by the observed patients as low [22].

All other studies (11 out of 13 studies) reported a successful outcome in terms of change in their measured outcome parameters:

Adherence to IP measures showed a positive change in all nine out of the nine studies in which it was examined.

SSI rates decreased after intervention in 5 out of the 6 studies in which it was examined. One study reported no significant change in SSI rate whereas successfully influencing all other outcome parameters (DDD, Cost of antibiotics, LOS, iA) [19]. Preinterventional SSI rates ranged from 28% as the maximum to 1.2% as the minimum [20, 24]. Postinterventional SSI rates were reported as 0% in 3 out of the 6 studies. The remaining 2 out of 6 studies reported postinterventional SSI rates ranging from 2.8% to 0.54%. SSI surveillance methods and SSI definitions were not reported. Due to a lack of further specification and missing information on sample sizes, pooling of the data was not possible and no further statistical analysis was performed.

Length of Stay (LOS) was reported in two studies stating a decrease after the intervention (Details: Supplement 2).

Cost of antibiotics Per Case was reported in two studies and a decrease in cost was stated in both (Details: Supplement 2). DDD/100 patient bed-days, reported in one study, showed a decrease [19].

### Implementation methods

#### Successful implementation methods

11 out of 13 studies reported success concerning the implementation of IP measures. Development of standardized guidelines was reported in 10 out of 11 studies (successful concerning iA in all seven studies, dSSI% in 5 out of 6 studies, LOS in 2 studies, cost of antibiotics in 2 studies, DDD in one study). Printed materials for staff and/or patients were considered in 9 studies reporting success (concerning iA in 5 out of 6 studies, dSSI% in all 4 studies, LOS in 1 study). Building of a Multidisciplinary Team was reported in 8 out of 11 studies reporting success (successful concerning iA in five studies, dSSI% 5 studies; LOS, cost of antibiotics, DDD in 1 study). Audits and Regular Meetings were reported in 7 out of 11 successful studies (successful concerning iA in four studies, dSSI% in 5 studies, LOS in 1 study). Personal lectures, personal trainings and other interactive methods were reported in 8 out of 11 studies (successful concerning iA in five studies, dSSI% in 4 studies; LOS, cost of antibiotics, DDD, surface area of hands missed in one study). Feedback was reported in 7 out of 11 studies (successful concerning iA in four studies, dSSI% in three studies). Online and electronic materials were used in 5 out of 11 studies (successful concerning iA in 4 studies, dSSI% in 3 out of 4 studies, LOS and cost of antibiotics in one study). Cost analysis and economic feedback were reported in 2 out of 11 studies (successful concerning iA in 1 study, dSSI% in 1 study). Verification tools for patient compliance were reported in 2 out of 11 studies (successful concerning iA 2 out of 3 studies, dSSI% 1 study). See Table 1 for graphic presentation.

Other methods reported in successful studies, none of which reported more than once, were: daily ward visits, spontaneous knowledge tests, online knowledge test as a follow-up, assessment of knowledge pre-intervention, town hall meetings, consulting service from exemplar external hospitals, installation of a contact system for (positively) screened patients, physician leadership encouragement, root-cause analyses for SSI cases, monitoring of antibiotics and antibiotics costs by pharmacists, change of patient admission process. One study reported employing a pre-existent standardized protocol for implementation (Johns Hopkins Nursing Evidence-Based Practice Model) [27].

#### Unsuccessful implementation methods

One study reported lack of success when using posters as a stand-alone implementation method with the goal of behavioral change in the OR (no significant change in adherence to promoted behavioral patterns) [26]. In the study, pre-interventional adherence to the proposed behaviors was overall already high (65%); but aspects that showed a low pre-interventional adherence showed no improvement either. One study reported a pre-study intervention with 12 months of hand hygiene education by posters resulting in persistent deficits in hand hygiene performance as a result [25].

One study reported a low level of compliance (22%) with preoperative antiseptic washings by the patient using printed information material for the patient and personal instructions [22].

#### Comparative studies

One study reported comparing two different implementation methods with the goal of iA to PAP guidelines: One study group, functioning as the control group, was given paper-based PAP guidelines, and in the interventional group, electronic PAP guidelines were installed into the order-entry system (accessible for the surgeon when ordering the antibiotics). The study reported better results for electronic-based implementation concerning iA, LOS and Cost Of Antibiotics. SSI% was not affected [19].

One study reported on a quality improvement campaign in the design of a cluster-randomized multicentric trial. A bundle of IP measures was proposed to a group of hospitals, out of which, randomly two groups were selected: an intervention group (78 hospitals) taking part in a QI campaign versus a control group (95 hospitals) not taking part in the QI campaign. The campaign provided implementation tools (QI methods) to the hospitals in the intervention group. The study reported higher adherence to the IP bundle in the intervention group utilizing the proposed implementation methods (see Supplement 2) as compared to the control group without access to the implementation materials [17, 18].

### Personnel Involved

**Fig. 3:**
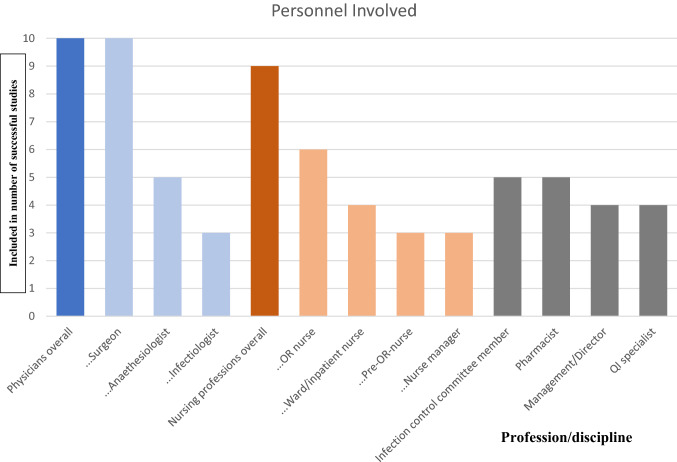
Personnel involved. *OR* operation room, *QI* quality improvement

In the included studies, it was not consistently stated if the personnel mentioned were primarily target audience or part of the implementers. We sub-summarized the specialties in one group each nevertheless (Fig. 3). Out of *n* = 11 studies reporting success, *n* = 10 were eligible for analysis of personnel involved. One study did not specify the involved personnel (“all staff of orthopedic department”) [25]. Involved personnel were assessed as specifically stated in the studies, no assumptions were made. The results are shown in Fig. 3.

### Study periods

7 out of 13 studies reported on a time period for the interventional period. Implementation time ranged from 10 days to 18 months. Single IP measures were implemented in 1.77 months on average (*n* = 3; range 10–90 days). Bundled IP measures were implemented in 9.31 months on average (*n* = 4; range 1.25–18 months).

## Discussion

Evidence for implementation methods of IP measures in orthopedics and trauma surgery is scarce. Nevertheless, we were able to identify some recurring implementation methods that seem to be valuable when planning IP improvement interventions. Interpersonal communication and interdisciplinary work seem to play a role in successful implementation. It, thus, seems recommendable to adhere to following methods as a standardized process when planning and executing an intervention with the aim of implementing IP measures in orthopedics and traumatology:

1. Forming of a multidisciplinary team including non-physician professions (with an emphasis on nursing professions)
2. Regular meetings of the team
3. Development of Standardized (Interdisciplinary) Guidelines
4. Sufficient resources for information printed and online/electronic; with possible software implementation
5. Personal Lectures and Training, Interactive Measures
6. Regular Feedback and Audits

These implementation methods address behavioral as well as cognitive levels; the multilevel *bundled* approach proves valuable for IP measures as well as implementation methods. The interdisciplinary approach has to be stressed as the danger of therapy-associated infection is an interdisciplinary, multifaceted problem. Our findings suggest the involvement of surgeons as primary stakeholders and *champions* of IP and implementation. Nursing professions should be given a forum as they carry out a big portion of the workload. On the other hand, the executive suite, management professions, economic and clinical leaders should be involved to stabilize planning, made easier by interdisciplinary agreeing on guidelines and best practice to estimate costs.

It is noteworthy that every study on the implementation of IP measures comprised by more than one implementation method reported clinical success. This means that, apart from economic considerations, as long as one *does* intervene—there will likely be success [36].

The data extracted in the review cannot provide a quantification of the importance of each single implementation method, but some of the included studies reported what the authors stressed as important factors for success. Multidisciplinary team approach and interdisciplinary consensus were mentioned in 6 out of 11 successful studies [21, 23, 24, 27–29]. Other aspects mentioned as impactful were personal training [19, 29], continuous training and feedback [24, 29], monthly analysis [21], active involvement of every member and physician leadership [21, 24] and patience [24]. Implementation methods reaching the cognitive level only (education by posters in this case) were not followed by improvement, which fits established implementation research [30]. The impression gained from the included studies is that there should be no separate entities in terms of *IP measure* and *implementation method* but rather a synthesis of *what and how*: The *how*-aspect of infection prevention seems to pose the greater obstacle for clinicians at times, accordingly clinical research on infection prevention should automatically also be researched on clinical implementation. The results go along well with current implementation science´s findings and theoretical models, many of which were published later than the studies included in this review [31, 32]. Beyond our own findings in orthopedics and trauma surgery, implementation science has gathered knowledge about change management in the health care sector. Five major domains were identified as being crucial for implementation: outer setting, for example the resources of a healthcare system; inner setting, for example, the structure of the ward where the implementation takes place; characteristics of the individuals involved; intervention characteristics; and the process of implementation [31]. According to our results, implementation measures used in trauma surgery so far focus on subdomains of the inner setting (teambuilding aspects) and the process of implementation itself (how to inform staff, measurement and feedback of results). Since implementation barriers are often based on problems with team communication, and departmental culture is a key factor for successful implementation [33], giving special attention to team building measures seems like a reasonable approach. Members must be aware of being a part of the implementation process [33]. Nevertheless, in future studies, additional domains or subdomains may be taken into consideration to enhance the overall effect and implementation success. Leadership and the support of management and middle management play an important role [31, 33, 34]. The legitimacy of the source may also influence implementation [31]. It seems to play a role if an intervention is initiated by a widely accepted entity. Following the Theory of planned behaviour by Ajzen, human behaviour depends on attitude, subjective norm and perceived behavioural control [35]. Thus, besides convincing the team members and conveying of content, etc., it is crucial to make the desirable conduct as easy and comfortable as possible for the staff. This may be another factor to increase compliance.

There are, however, limitations to the recommendations both for formal reasons and with regard to contents. Primarily, there are concerns about the quality of the included studies.

The MINORS score average is rather low with a probably moderate-to-high risk of bias. After quality assessment via MINORS scores and Risk of Bias evaluation, we decided to keep all studies in the review despite the disparate range of 4–13 out of 16 points, as there is no clear cut-off suggestion on MINORS scores found in the literature and we decided to rather discuss the findings in a cautious way. Since the results of the studies reporting success are rather homogeneous, we did not see an additional benefit for the interpretation by weighing the individual Risk of bias of the included studies. Interventions designed to achieve behavioural change or change in overall culture of a workplace surrounding are inherently biased as in that every possible parameter is manipulated towards achieving the desired outcome, only the outcome itself should in the end be measured neutrally. The studies not showing successful intervention showed a clear enough difference in terms of implementation methods used not to conclude a heterogenity of results. Funding of the included studies was not consistently reported with only 5 out of 13 studies claiming no external funding or public funding. The inconsistency reporting of data made data pooling unfeasible. Reported improvements, therefore, have to be processed cautiously. In regard to contents, it has to be noted that rather frequently there was no in-depth description of implementation methods. There is, for example, no universal definition of *interactive training* or *personal training* or *teaching* [32, 36]. This terminology was inconsistent throughout the studies, which has been criticized in implementation research before; therefore, we had to sub-summarize these methods as one entity, though it might well play an important role how exactly the interpersonal work was designed [37]. Occasionally, the clear separation of an IP measure and an implementation method was difficult (which leads to the above stated conclusion that generally they should not be considered separate entities). Additionally, it was not possible to determine which of the implementation methods included in the successful studies had the biggest impact, which is a known problem, but it was possible to approximate by analyzing the frequency of use [36, 38]. Addressing the problems encountered (and possible solutions) while implementing IP measures could also be underreported and possibly biased. One author stated that they underestimated the “complexity of hospital structures and surgical outcome measures” as well as the “variability of perceived role of disciplines” [28]. Another author faced economic problems as the implementation cost per patient seemed to exceed the cost of one SSI [27]. One study reported limited resources for the pharmacy department as advisors to the surgical department as a hurdle [19]. Two authors reported a feedback from implementers stating a “lack of physician buy-in and staff resistance”, economic aspects (cost of antibiotics/antiseptic agents), logistics of preoperative IP measures (antiseptic washings) and “a perceived lack of evidence” were the main problem fields [17, 18]. One hospital representative selected for a QI campaign stated that “SSIs are not a problem” [17, 18]. One study discussed that adherence and improvement thereof would be measurable, but sample sizes to be underpowered to demonstrate an effect on SSI% [23].

Hospital structure and structural organization of health care might be difficult to assess when transferring implementation methods internationally or inter-hospital-wise (e.g., responsibilities and educational standards of different professional groups), but general principles of communication and interdisciplinary might work universally. Nevertheless, proper description of the inner and outer setting as an important aspect for implementation is advisable for future publications: *what has worked in a setting similar to ours?* Least evidence on how to implement IP measures was found for the collective of trauma surgery patients, who are especially prone to infectious complications, but the findings might be transferable—the realities of the health care system suggest that a substantial fraction of the workers are involved in the treatment of both patient cohorts. Especially the “semi-elective” trauma surgery patients (i.e., those waiting for definitive fracture fixation) can surely be introduced to the measures and methods effective in elective surgery. The included studies focused on SSIs, while it would be possible that other types of hospital-acquired infections were affected. Finally, economic calculations concerning implementation resources (training, learning, materials, and personnel) are tough to carry out, but would provide helpful arguments for planners of IP improvement campaigns for getting stakeholders on board.

There might be important insight on the topic of implementation of IP measures from other clinical fields which was not considered in this study due to the limitation to orthopedics and traumatology [39–50].

## Conclusion

Although evidence was scarce and quality-inconsistent, we found that adhering to a set of implementation methods focusing on interdisciplinary and interactive/interpersonal work might be an advisable strategy when planning IP improvement interventions in orthopedics and traumatology.

## Electronic supplementary material

Below is the link to the electronic supplementary material.

Supplementary file1 (DOC 36 kb)

Supplementary file2 (DOC 80 kb)

Supplementary file3 (DOC 46 kb)

Supplementary file4 (DOCX 55 kb)
