# Correction to: Implementation methods of infection prevention measures in orthopedics and traumatology – a systematic review

**DOI:** 10.1007/s00068-021-01610-6

**Published:** 2021-03-09

**Authors:** Benedikt Marche, Meike Neuwirth, Christiane Kugler, Bertil Bouillon, Frauke Mattner, Robin Otchwemah

**Affiliations:** 1 Dept. of Orthopedics, Trauma Surgery and Sports Medicine, University Witten/Herdecke, Cologne Merheim Medical Center, Ostmerheimer Str. 200, 51109 Cologne, Germany; 2 Institute for Hygiene, Cologne Merheim Medical Center, University Hospital University Witten/Herdecke, Ostmerheimer Str. 200, 51109 Cologne, Germany; 3 Bereich Pflegewissenschaft, Albrecht-Ludwigs-Universität Freiburg, Freiburg, Germany

## Correction to: European Journal of Trauma and Emergency Surgery 10.1007/s00068-020-01477-z

The original version of this article unfortunately contained a mistake.

The affiliations 1 and 2 were incorrect. The correct information is given below.

1 Dept. of Orthopedics, Trauma Surgery and Sports Medicine, University Witten/Herdecke, Cologne Merheim Medical Center, Ostmerheimer Str. 200, 51109 Cologne, Germany.

2 Institute for Hygiene, Cologne Merheim Medical Center, University Hospital University Witten/Herdecke, Ostmerheimer Str. 200, 51109 Cologne, Germany.

The original article has been corrected.

