# Supplementary material for: Implementation methods of infection prevention measures in orthopedics and traumatology – a systematic review
Source: Eur J Trauma Emerg Surg. 2020 Sep 10;47(4):1003–13. doi: 10.1007/s00068-020-01477-z (PMC8321980; doi:10.1007/s00068-020-01477-z)
Supplement: Supplementary file 1 — Supplementary file1 (DOC 36 kb) [file 68_2020_1477_MOESM1_ESM.doc]

Table Of Excluded Studies After Full-Text-Check

| **AUTHOR/YEAR/TITLE** | **REASONS FOR EXCLUSION AFTER FULL-TEXT-ANALYSIS** |
| --- | --- |
| *Schneeberger PM*, 2002 „Surveillance as a starting point to reduce surgical-site infection rates in elective orthopaedic surgery“ [39] | Surveillance Project only, no direct implementation process described |
| *Bratzler DW, 2006* „The Surgical Infection Prevention and Surgical Care Improvement Projects: National Initiatives to Improve Outcomes for Patients Having Surgery“ [42] | Report on nationwide QI campaign, IP measures described, baseline parameters given, but no report on intervention, implementation methods and outcome |
| *Aboelela SW, 2007* „Effectiveness of bundled behavioural interventions to control healthcare-associated infections: a systematic review of the literature“ [36] | Review on implementation methods for IP measures, patient cohort/clinical discipline not orthopedics and/or traumatology |
| *Rosenberger LH, 2011* „The Surgical Care Improvement Project and Prevention of Post-Operative Infection, Including Surgical Site Infection“ [48] | Report on Proposed Bundle of IP measures for QI campaign, no implementation methods stated |
| *Barchitta M*, 2012 „Prevalence of surgical site infections before and after the implementation of a multimodal infection control programme“ [40] | Patient cohort included 20 different surgical departments, cohort of orthopedics and traumatology could not be identified |
| *Jordan CJ, 2012* „Comprehensive Program Reduces Hospital Readmission Rates After Total Joint Arthroplasty“ [47] | Interventional study of IP-measure-bundle; implemenation methods of IP measures not reported |
| *Sartini M*, 2013 „Improving environmental quality in an operating room: clinical outcomes and economic implications“ [41] | Interventional study of IP-measure-bundle; implemenation methods of IP measures not reported (dixit „Quality Improvement Scheme“) |
| *Ryan SL*, 2014 „A standardized protocol to reduce pediatric spine surgery infection: a quality improvement initiative“ [44] | Interventional study of IP-measure-bundle; implemenation methods of IP measures not reported |
| *Navone P, 2015* „From the risk analysis to the development of interventions and training for the prevention and control of healthcare associated infections. The experience of G. Pini Orthopedic Institute“ [45] | Republication of already included study (Nobile M, 2014, „Reinforcing good practice: Implementation of guidelines at hospital G. Pini“) |
| *Morris AJ, 2015* „A new surgical site infection improvement programme for New Zealand- early progress“ [46] | Interventional study of IP-measure-bundle; implemenation methods of IP measures not reported |
| *Metsemakers WJ*, 2017 „Prevention of fracture-related infection: a multidisciplinary care package“ [43] | Review of fracture-/trauma-related hygiene measures; stresses importance of multidisciplinary approach; implemenation methods of IP measures not reported |
