# Supplementary material for: Implementation methods of infection prevention measures in orthopedics and traumatology – a systematic review
Source: Eur J Trauma Emerg Surg. 2020 Sep 10;47(4):1003–13. doi: 10.1007/s00068-020-01477-z (PMC8321980; doi:10.1007/s00068-020-01477-z)
Supplement: Supplementary file 2 — Supplementary file2 (DOC 80 kb) [file 68_2020_1477_MOESM2_ESM.doc]

Supplement 3: Matrix of Included Studies Ordered by Publication Date

PAP: Perioperative Antibiotic Prophylaxis; pre-OP: preoperative; OR: Operation Room; UTI: Urinary Tract Infection; SSI%: Surgical Site Infection Rate; LOS: Length Of Stay; A/C: Adherence/Compliance; DDD: Defined Daily Doses; QI: Quality Improvement; IP: Infection Prevention;

| **AUTHOR/YEAR/**  **STUDY TYPE** | **PATIENT**  **COHORT,**  **Sample Size** | **IP MEASURES** | **WAY OF IMPLEMENTATION**  **TIMEFRAME OF IMPLEMENTATION** | **PERSONNEL**  **INVOLVED** | **OUTCOME PARAMETERS** | **EFFECT**  **(OUTCOME)** | **TIME-**  **FRAME*** |
| --- | --- | --- | --- | --- | --- | --- | --- |
| **Douglas**  ***J Qual Clin Practice 2001***  **[20]**  Interventional  prospective cohort study | Elective Orthopedics  sample size not stated | **Bundle**  - Correct PAP  - Risk stratification/risk factor assessment in patients pre-OP  - Preoperative antiseptic washing  - OR: technical and behavioural: pulsed irrigation, gloving techniques, correct gown/mask, reducing OR traffic  - Improved wound care | - Multidisciplinary team  - Regular meetings, audits  - Standardized guidelines for antibiotics, UTI management, wound care  - Printed materials (Audit tools, flow charts)  - Protocol for patient admission process,  Patient selection criteria development  - Personal training of nurses/staff | - Surgeon  - Infectiologist  - OR manager  - Nurse manager  - Area Health Service Director  - Director of Clinical Service | SSI%  Length of stay (d) | Positive (SSI 28%-> 0%)  Positive (LOS 13.9d → 9.3d) | Not stated |
| **Queiroz**  ***Braz J Infect Dis 2005***  **[23]**  Interventional  prospective cohort study | Elective orthopedics  and  traumatology  n=299 patients/  cases | **Single**  Correct PAP  - Indication  - Choice  - Timing  - Dosage  - Redosing  - Postoperative discontinuation | - Multidisciplinary team  - Standardized guidelines for antibiotics  - Lectures to residents  - Interdisciplinary discussion of rationale on daily ward visits  - Surgeon signs antibiotic order form before surgery  - Monitoring of PAP/antibiotics by pharmacist | - Surgeon  - Pharmacist  - Infection control committee | SSI%  Frequency of appropriateness of PAP (%) (A/C)  Cost of Antibiotics/case  DDD of antibiotics/100 bed-days | Positive (SSI 4.0% → 2.8%)  Positive (A/C 3.3% → 50%)  Positive (16.6$ → 6.6$)  Positive (DDD 8.53 → 7.9) | 3 months |
| **Macdonald**  ***Ann R Coll Surg England 2006***  **[25]**  Interventional  prospective cohort study | Orthopedics  n=55 staff | **Single**  Hand hygiene performance | - Spontaneous practical testing of hand hygiene and result presentation on ward  - Reeducation by poster  - Personal re-testings with feedback  (unsuccessful poster campaign prior to study) | Not specified  (mixed staff of Orthopedic Dept.) | Surface area of hands missed (%) | Positive  (7.8% → 2.3% surface area of hands missed) | Not stated |
| **McCahill**  ***Arch Surg 2007***  **[28]**  Interventional  prospective cohort study | Elective orthopedics  n=317 patients/  cases | **Single**  Correct PAP:  - Choice  - Timing  - Postoperative discontinuation | - Multidisciplinary team  - Standardized guidelines for antibiotics  - Regular meetings, audits, error identification  - Small task forces, defined responsibilities  - Regular reporting to directors/managers  (chiefs of surgery, quality committee, board of trustees)  - Written orders on PAP by surgeon pre-OP with confirmation by nurse (time-out)  - Time-out confirmation of PAP: Confirmation, responsibility and documentation of PAP by anesthesiologist  - Physician education  - Standardized post-OP-order forms  - Feedback (reporting SSI% to surgeon) | - Surgeon  - Anesthesiologist  - Pre-OR nurse  - OR nurse  - Ward nurse  - Pharmacist  - Infection control committee member  - QI-expert | Frequency of appropriateness of PAP in %  (A/C) | Positive  (A/C Choice of PAP  72% → 84%,  A/C Timing of PAP  98% → 98%;  A/C Post-OP discontinuation of PAP  55% → 87%) | 9 months |
| **Mackain-Bremner, A.**  ***Ann R Coll Surg England 2008***  **[26]**  Interventional  prospective study | Elective  orthopedics  n=82 staff | **Bundle**  - Correct PAP  - Multi-faceted behavioural IP measures in OR, i.e.:  - removal/covering of jewlery  - ears, nose, hair covered  - shirts tucked in  - gloves left untouched  - Alcoholic agent for operative skin preparation | - Posters in scrub area of OR  („strategic places“) | - Surgeon  - Anesthesiologist  - OR nurse  - Anesthesia nurse  - Industry representatives  - Porters  - Consultants  - Medical students  - House officers | A/C to/with behavioural aspects (%) | Not successful  (no improvement) | 10 days |
| **Rosenberg**  ***J Bone J Surg Am 2008***  **[50]**  Interventional  prospective cohort study | Elective orthopedics  n=319 patients/  cases | **Single**  Correct PAP  - Choice  - Timing  - Dosage | - Standardized guidelines for antibiotics  - Standardized (printed) surgical time-out-checklist  - Assignment of responsibility | - Surgeon  - Anesthesiologist  - OR nurse | Frequency of appropriateness of PAP in %  (A/C) | Positive  (A/C 65% → 96.8%) | 7 weeks |
| **Nobile**  ***Injury 2014***  **[29]**  Interventional  prospective cohort study | Elective orthopedics  n=124 patients/  cases | **Single**  Correct PAP:  - Indication  - Choice  - Timing  - Dosage  - Postoperative discontinuation | - Multidisciplinary team  - Standardized guidelines for antibiotics  - Regular meetings  - Assessment of current knowledge  - Training events (ubiquitary)  - Printed Materials  (Pocket antibiotics guideline distribution)  - Registration of antibiotics  - Error reporting system  - Feedback: Interdisc. discussion of errors with involved personnel, result presentation  - Events on SSI-prevention for nursing professions  - Electronic materials  - Online reassessment of knowledge (online surveys) | - Surgeon  - Infectiologist  - Infection control committee (nurse)  - Public health specialist  - Health management  - Anesthesiologist  - OR nurse  - Pharmacist | Frequency of appropriateness of PAP in %  (A/C) | Positive  (A/C 36% → 63%) | 2 months |
| **Yang**  ***PloS One 2014***  **[19]**  prospective, comparative interventional study, non-randomized | Orthopedic  n=717 patients/  cases  (n=357  cases as  control group  vs.  n=360  cases as  interventional group) | **Single**  Correct PAP:  - Choice  - Timing  - Postoperative discontinuation | - Standardized guidelines for antibiotics  - Interventional group:  Installation of Online Antibiotic Guideline into surgeons PAP Order-Entry System  (with direct data collection and surveillance)  - Control group:  Distribution of paper based guidelines | Surgeon | Frequency of appropriateness of PAP in %  (A/C)  Length of stay (d)  Cost of Antibiotics/Case  SSI% | Interventional group:  Superior  - A/C (70% vs. 11%)  - LOS (7.0±4.31 days → 2.54±1.57 days)  - Cost of Antibiotics/Case (1700¥ vs 3500¥)  No difference in SSI% (10% in interventional and control group) | 2  months |
| **Kapadia**  ***Surg Technol Int 2015***  **[22]**  retrospective study | Elective orthopedics  n=4751 patients | **Single**  Preoperative antiseptic washing by patient | - Printed materials (instruction sheet for patient)  - Compliance testing process  (Patient is controlled at admission)  - Questionnaire (oral) at admission day  - Promotion of antiseptic washing by ancillary staff | - Surgeon  - Ancillary staff  (medical personnel not specified, admission personnel) | A/C (patient) (%)  (SSI%) | Not successful  (A/C 22% to washings rated as „low“ by the study author)  (lower in compliant patients, 0.6% vs. 1.9% for non-compliant) | Not stated |
| **Khodyakov**  ***BMJ Qual Saf***  ***2015***  and  **Schneider**  ***Implement Sci 2017***  **[17, 18]**  cluster-randomized trial | Elective orthopedics  n=173 hospitals  (n=78 hospitals as interventional  group  vs.  n=95 hospitals as control group) | **Bundle**  - Correct PAP  - Universal S. aureus screening  - Selective decolonisation  - Correct hair removal (clipping)  - Preoperative antiseptic washing  - Alcoholic agent for operative skin preparation | *Provided Implementation Methods/Materials (10) for Hospitals in Intervention group:*  *-* Encouragement for feedback  - Encouragement to form multidisciplinary team  - Encouragement to monitor/test changes  (- Standardized guidelines)  *-* printed materials  (patient handouts, how-to-guide, evidence materials, improvement tools, business case)  - online materials  (patient handouts, how-to-guide, evidence materials, improvement tools, business case)  *-* Interactive, electronic/online resources  (webinars, website, in-person presentations, town hall meetings, site visits, consultation from exemplar hospitals)  *Control Group:*  *No Implementation Methods/Materials provided* | - Surgeon  - Nurse  - OR personnel  - QI expert | IP bundle adoption  (A/C)  A/C with each bundle component (IP measure)  Usage rate of each of 10 resources provided  A/C with QI/implementation methods  Correlation between A/C with implementation methods and A/C with IP measures | Positive (intervention group): 55% complete IP bundle uptake  Positive  Most frequently used implementation methods:  webinars/electronic communication: 85%)  52% implementation plan  90% forming of multidisc. implementation team  36% tests of change  Positive (Odds Ratio 2.53) | 6 months |
| **Mori**  ***Int J Orthop Trauma Nurs 2015***  **[27]**  Interventional  prospective cohort study | Elective orthopedics  sample size not stated | **Bundle**  - Universal S.aureus screening 2 weeks before surgery (nasal)  - Selective decolonisation of S.aureus carriers (nasal)  - Preoperative antiseptic washing | „Johns Hopkins Nursing Evidence-Based Practice Model“  - Multidisciplinary team  - Standardized guidelines for antibiotics etc.  - Regular Meetings  - Cost analysis presentation to management  - Printed information materials (patient education documents, preparation checklist)  - Installation of new screening process  - Verification tools for patient compliance  - Construction of organisational/structural process to make decolonisation material available to patient  - Contact system for reaching positively screened patients  - Interactive education class for patients  - Telephone reminder for patients concerning decolonisation  - Feedback (SSI% to staff)  - Monitoring of costs | - Surgeon  - Infection Control Comittee member  - Nursing manager  - Infectiologist  - Outpatient manager  - Pharmacist  - Preadmission nurse  - Laboratory manager | SSI% | Positive  (SSI% 5.3% → 0%) | 6  months |
| **Shea**  ***J Pediatr Orthop 2015***  **[24]**  Interventional  prospective cohort study | Elective  (pediatric)  orthopedics  sample size not stated | **Bundle**  - Universal S.aureus screening  - Selective decolonisation  - OR: Behavioural and technical IP measures: Correct gowns, hand hygiene, sterilization, skin preparation, OR traffic, room cleaning  - improved wound care  - Identifcation of patient-related and environment-related risk factors preoperatively | - Multidisciplinary team  - Regular meetings  - Standardized guidelines/protocols  - Preoperative clinic staff installed  - Educational programs for all OR personnel  - Posters in lounges and meetings  - electronic materials: email updates  - Improvement of interdisciplinary communication, encouragement for input  - Printed materials (case preference cards)  - feedback  - physician leadership encouragement | - Surgeon  - Infectious desease comittee members  - Administration  - OR nurse  - Inpatient nurse  - Sterilization/Central instrument processing  - Anesthesiologist  - Service staff  - Engineering staff | SSI% | Positive  (SSI% 1.2% → 0.54%) | 18 months |
| **Schriefer, J**  ***Int J Orthop Trauma Nurs 2017***  **[21]**  Interventional  prospective cohort study | Elective (pediatric) orthopedics  n=541 patients/  cases | **Bundle**  - Correct PAP  - Preoperative nutrition management  - Universal Decolonisation (nasal)  - Preoperative antiseptic washing  - Normothermia  - Urinary catheter discontinuation <48h  - Improved wound care  - MRSA-surveillance | - Multidisciplinary team  - Standardized guidelines  - Printed materials (e.g. dosing chart)  - Regular meetings/audits  - Feedback/result presentations  - Risk stratification of patients  - Electronic materials: Incorporation of PAP guidelines in electronic patient record system  - Compliance tracking  - Printed instruction materials for patients (e.g. CHG-wiping technique)  - Documentation A/C with preoperative antiseptic washing  - Root-Cause-Analysis for each SSI-case  (printed case review form) | - Surgeon  - Anesthesiologist  - OR nurse  - Inpatient nurse  - Pre-OR nurse  - Pharmacist  - Infectiologist  - Radiologist  - QI-expert | A/C with complete bundle in %  and  compliance with each component in %  SSI % (yearly) | Positive  Positive  (SSI% 4% → 3,2% → 0%) | 12 months |

*Timeframe for interventional period, „implementation time“
