# Supplementary material for: Implementation methods of infection prevention measures in orthopedics and traumatology – a systematic review
Source: Eur J Trauma Emerg Surg. 2020 Sep 10;47(4):1003–13. doi: 10.1007/s00068-020-01477-z (PMC8321980; doi:10.1007/s00068-020-01477-z)
Supplement: Supplementary file 3 — Supplementary file3 (DOC 46 kb) [file 68_2020_1477_MOESM3_ESM.doc]

| **AUTHOR/YEAR** | Clearly stated aim | Inclusion of consecutive patients | Prospective  collection of data | Endpoints appropriate  to the aims of study | Unbiased assessment  of the study endpoint | Follow-up period appropriate  to the aim of the study | Loss to follow up  less than 5% | Prospective calculation  of the study size | An adequate control group | Contemporary groups | Baseline equivalence  of groups | Adequate statistical analyses | **MINORS Score**  (max. 16/16 for  non-comparative,  max. 24/24 for comparative studies) |
| --- | --- | --- | --- | --- | --- | --- | --- | --- | --- | --- | --- | --- | --- |
| Douglas 2001 | 2 | 0 | 2 | 1 | 0 | 0 | 0 | 0 | - | - | - | - | **5/16** |
| MacDonald 2006 | 2 | 2 | 2 | 1 | 0 | 0 | 2 | 0 | - | - | - | - | **9/16** |
| MacKain-Bremner 2008 | 2 | 2 | 2 | 2 | 1 | 1 | 2 | 0 | - | - | - | - | **12/16** |
| McCahill 2007 | 2 | 2 | 2 | 2 | 0 | 2 | 2 | 0 | - | - | - | - | **12/16** |
| Mori 2015 | 2 | 0 | 1 | 1 | 1 | 2 | 2 | 0 | - | - | - | - | **9/16** |
| Nobile 2014 | 2 | 0 | 2 | 2 | 1 | 2 | 1 | 0 | - | - | - | - | **10/16** |
| Queiroz 2005 | 1 | 2 | 2 | 1 | 0 | 2 | 2 | 0 | - | - | - | - | **10/16** |
| Rosenberg 2008 | 2 | 2 | 2 | 2 | 2 | 2 | 1 | 0 | - | - | - | - | **13/16** |
| Schriefer 2017 | 2 | 1 | 2 | 2 | 1 | 1 | 0 | 0 | - | - | - | - | **9/16** |
| Shea 2015 | 1 | 0 | 2 | 1 | 0 | 0 | 0 | 0 | - | - | - | - | **4/16** |
| Yang 2014 | 2 | 2 | 2 | 2 | 2 | 0 | 0 | 0 | 2 | 2 | 2 | 2 | **18/24** |
| Schneider/  Khyodyakov 2017/2015 | - | - | - | - | - | - | - | - | - | - | - | - | N/a (Cluster-Randomized Trail) |
| Kapadia 2015 | (2) | (2) | (0) | (2) | (1) | - | - | (0) | - | - | - | - | N/a (Retrospective Study) |
