# Supplementary material for: Implementation methods of infection prevention measures in orthopedics and traumatology – a systematic review
Source: Eur J Trauma Emerg Surg. 2020 Sep 10;47(4):1003–13. doi: 10.1007/s00068-020-01477-z (PMC8321980; doi:10.1007/s00068-020-01477-z)

| STUDY/YEAR | RISK OF BIAS DOMAIN | | | | | | | OVERALL  RISK OF BIAS |
| --- | --- | --- | --- | --- | --- | --- | --- | --- |
|  | BIAS DUE TO CONFOUNDING | BIAS DUE TO SELEC-TION OF PARTICIPANTS | BIAS IN CLASSIFICATION OF INTERVENTION | BIAS DUE TO DEVIATION FROM INTERVENTIONS | BIAS DUE TO MISSING DATA | BIAS IN MEASUREMENT OF OUTCOMES | BIAS IN SELECTION OF REPORTED RESULTS |  |
| Douglas  2001 | 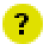 | 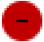 | 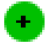 | 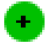 | 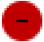 | 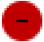 | 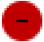 | 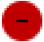 |
| Queiroz  2005 | 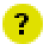 | 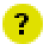 | 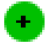 | 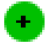 | 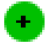 | 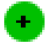 | 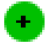 | 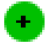 |
| Macdonald 2006 | 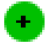 | 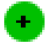 | 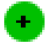 | 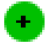 | 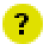 | 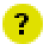 | 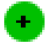 | 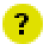 |
| McCahill  2007 | 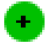 | 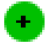 | 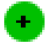 | 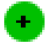 | 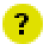 | 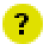 | 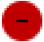 | 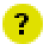 |
| Mackain-Bremner 2008 | 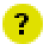 | 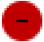 | 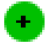 | 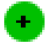 | 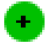 | 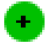 | 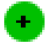 | 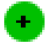 |
| Rosenberg 2008 | 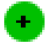 | 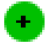 | 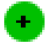 | 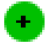 | 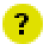 | 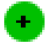 | 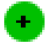 | 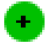 |
| Nobile  2014 | 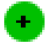 | 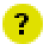 | 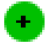 | 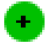 | 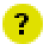 | 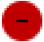 | 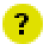 | 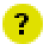 |
| Yang  2014 | 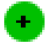 | 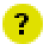 | 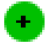 | 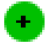 | 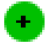 | 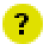 | 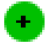 | 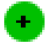 |
| Mori  2015 | 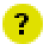 | 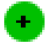 | 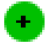 | 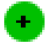 | 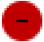 | 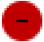 | 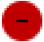 | 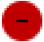 |
| Shea  2015 | 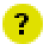 | 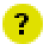 | 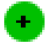 | 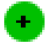 | 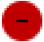 | 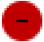 | 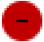 | 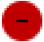 |
| Kapadia  2015 | 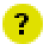 | 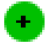 | 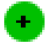 | 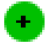 | 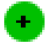 | 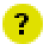 | 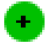 | 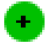 |
| Schriefer  2017 | 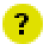 | 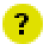 | 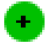 | 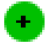 | 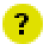 | 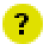 | 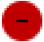 | 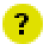 |

Table xx. Risk Of Bias Assessment Of Included NRS

NRS: non-randomized studies;
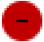
 = high risk of bias;
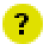
= unclear risk of bias;
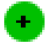
= low isk of bias.


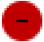

Supplement: Supplementary file 4 — Supplementary file4 (DOCX 55 kb) [file 68_2020_1477_MOESM4_ESM.docx]
